# Ribosome dynamics at the conserved PGP motif governs 2A peptide-bond-skipping efficiency

**DOI:** 10.1093/nar/gkag875

**Published:** 2026-09-08

**Authors:** Xincheng Wu, Saori Uematsu, Yan Wu, Shu-Bing Qian

**Affiliations:** Division of Nutritional Sciences, Cornell University, Ithaca, NY 14853, United States; Division of Nutritional Sciences, Cornell University, Ithaca, NY 14853, United States; Division of Nutritional Sciences, Cornell University, Ithaca, NY 14853, United States; Division of Nutritional Sciences, Cornell University, Ithaca, NY 14853, United States

## Abstract

Viral 2A oligopeptides drive an unusual ribosome recoding event in which peptide-bond formation fails at a conserved PG↓P motif, producing two discrete proteins without canonical termination. Despite decades of study, the molecular basis of 2A-mediated peptide-bond skipping remains poorly understood. Here, we combine quantitative 2A reporters with high-resolution ribosome profiling to interrogate ribosome dynamics at the core 2A sequences. We identify a pausing event at the terminal proline codon of the PGP motif that functions as a kinetic decision point: ribosome dwell time at this site inversely correlates with skipping efficiency. Increasing nascent chain flexibility by inserting linkers immediately upstream of the 2A sequence reduces ribosome occupancy at the terminal proline codon and enhances peptide-bond skipping. Strikingly, amino acid repeats positioned distally upstream also modulate 2A activity, indicating long-range coupling between nascent chain properties outside of the ribosome and the peptidyl transferase center inside the ribosome. In particular, hydrophobic residues potently suppress skipping, an effect that can be rescued by extending flexible segments within the peptide exit tunnel. Together, our findings support a model in which nascent chain features—beyond the core 2A motif—dynamically tune ribosomal recoding efficiency through co-translational feedback into the catalytic center.

## Graphical Abstract

**Graphical Abstract:**
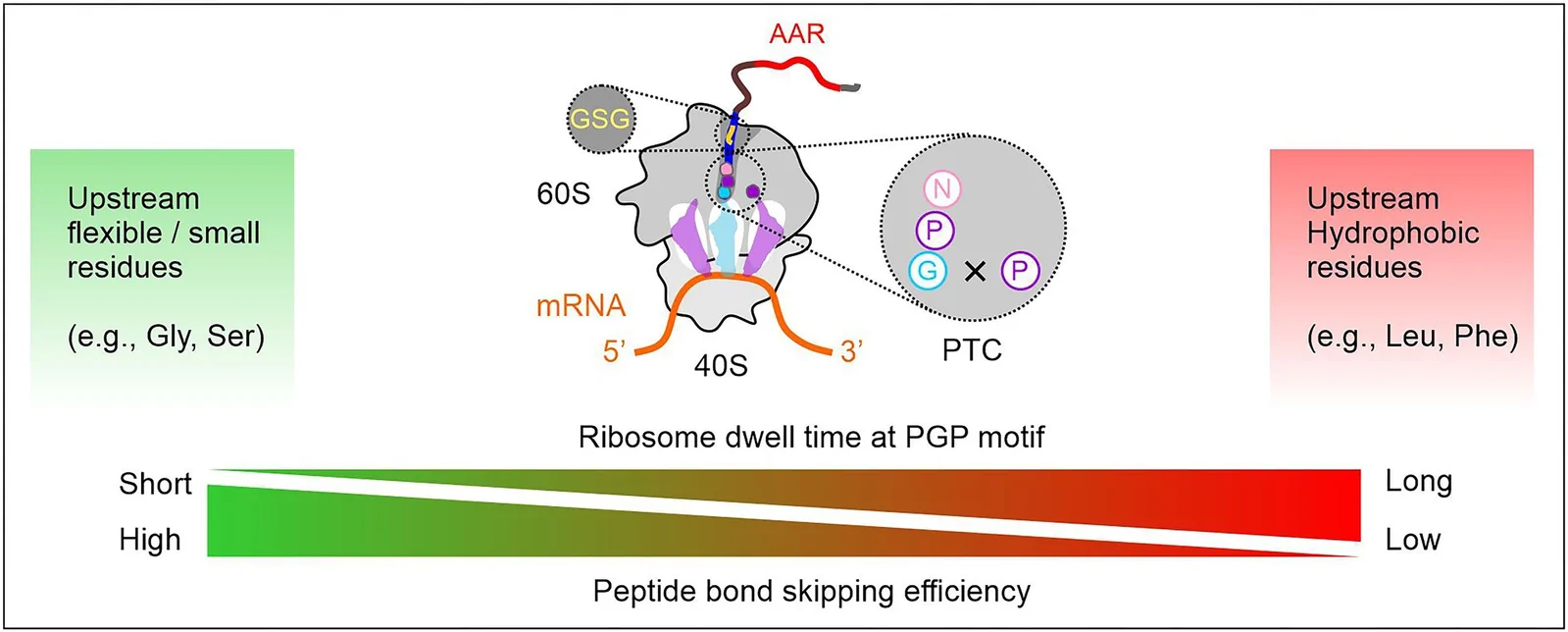

## Introduction

2A peptides are short viral elements (typically 18–22 amino acids) that mediate co-translational peptide-bond skipping within the ribosome [1–3]. First identified in foot-and-mouth disease virus, 2A sequences retain their activity when transferred into heterologous protein [1, 4]. Incorporation of 2A elements enables the expression of multiple proteins from a single open reading frame with near-stoichiometric ratios [5–7]. Owing to their robustness and modularity, 2A peptides are widely used in gene therapy, genome engineering, and recombinant protein production [8, 9]. Despite their broad utility, the molecular determinants governing 2A-mediated skipping efficiency remain incompletely understood.

Mechanistically, 2A peptides induce the failure of peptide-bond formation at a conserved PG↓P motif without canonical termination [2, 10]. The skipping event (indicated by ↓) yields an upstream peptide ending in Pro–Gly and a downstream peptide beginning with Pro [10]. Mutational analyses have defined a core octapeptide motif, -D(V/I)ExNPG↓P, as essential for the “stop-carry-on” activity [11]. Notably, sequences encoding upstream residues modulate skipping efficiency, implicating a crucial role of the nascent chain within the peptide exit tunnel in this recoding event [12]. A recent structural study suggested that interactions between the 2A motif and the peptide exit tunnel alter the conformation of the peptidyl transferase center (PTC), thereby inhibiting peptide-bond formation [13]. However, because the terminal Pro codon was replaced with a stop codon in these structures, how A-site tRNA-Pro accommodation is coupled to nascent chain release from the P-site remains unresolved.

A characteristic feature of the core 2A motif is the two proline codons flanking a glycine codon. Given that proline is an intrinsically poor substrate for peptide-bond formation, ribosome pausing at this motif may contribute to the failed peptide-bond formation [14–17]. In this framework, the ribosome dwell time at the PGP motif could determine whether peptide-bond formation proceeds or is bypassed. In theory, prolonged ribosome dwell time at the PGP motif could increase the chance of peptide-bond formation. Indeed, peptide-bond skipping barely occurs during translation of poly(Pro), a kinetically slow decoding process. Therefore, additional mechanisms likely contribute to the failed peptide-bond formation between the P-site glycine and the A-site proline. Empirically, insertion of flexible linkers (e.g. GSG) upstream of the 2A sequence enhances skipping efficiency, although the underlying mechanism remains unclear [7, 18–20]. The current view is that these upstream residues modulate PTC conformation, thereby hindering peptide-bond formation [13, 21]. Alternatively, increased flexibility may facilitate nascent chain movement within the exit tunnel, promoting A-site tRNA-Pro translocation and continuation of elongation. How upstream sequence context shapes ribosome kinetics at the PGP motif—and whether site-specific ribosome pausing dictates skipping efficiency—remains an open question.

Here, we apply high resolution ribosome profiling to interrogate ribosome dynamics at the porcine teschovirus-1 2A (P2A) sequence. We show that ribosome occupancy at the terminal Pro codon of the PGP motif inversely correlates with peptide-bond-skipping efficiency. Using natural amino acid repeats, we further demonstrate that upstream hydrophobic regions outside the ribosome suppress P2A activity. These findings establish a framework in which nascent chain properties broadly couple ribosome dynamics to translational recoding.

## Materials and methods

### Cell culture

HEK293 and Lenti-X 293T cells were maintained in Dulbecco’s modified Eagle’s Medium (Corning, 10-013-CV) supplemented with 10% fetal bovine serum (Sigma, 12306C) at 37°C in a humidified incubator with 5% CO₂.

### Lentiviral short hairpin RNAs

Short hairpin RNAs (shRNAs) targeting human EIF5A were designed based on sequences from The RNAi Consortium (https://portals.broadinstitute.org/gpp/public/) as follows: EIF5A: 5′-GCCATGTAAGATCGTCGAGAT-3′. A scrambled control sequence was 5′-AACAGTCGCGTTTGCGACTGG-3′. Target sequences were cloned into the DECIPHER pRSI9-U6-(sh)-UbiC-TagRFP-2A-Puro vector (Cellecta, CA). Lentiviral particles were produced in Lenti-X 293T cells (Clontech), and virus-containing supernatants were collected 48 h post-transfection and filtered. Target cells were infected for 48 h prior to selection with puromycin (1–2 μg/ml).

### Plasmid construction

Amino acid-rich regions were surveyed by scanning all the possible 20-codon sliding windows from the custom human transcriptome. The custom transcriptome was generated based on the reference genome and annotations obtained from Ensembl using human release 109 (GRCh38.p14). Protein-coding genes were extracted, and a single transcript was selected for each gene to the following procedure. For each gene, the transcript with the highest APPRIS score was initially selected. If the selected transcripts have several isoforms with the same APPRIS scores, the transcript with the longest coding sequence was included in the custom transcriptome. Candidate repeats were ranked by the frequency of occurrence of individual amino acid, and two top-ranked sequences were fused to generate a 40-codon insert, with the exception of Trp-, Phe-, and Met-rich regions, which rarely form extended tandem repeats. Concatenating two 20-codon repeats results in higher frequency of the target amino acid than that found in natural 40-codon repeats. A length of 40 codons is considered because the ribosomal exit tunnel spans 30–40 amino acid residues. This design would ensure that any observed effects on skipping are attributed to nascent chain properties outside the tunnel rather than to sequences still residing within it.

Amino acid-rich inserts (150 bp) were ordered as single-stranded oligos from Integrated DNA Technologies (IDT), containing a 120-bp amino acid-rich region flanked by 15 bp overlaps at each end. The 3×FLAG-miRFP670-P2A-EGFP-3×HA sequences were cloned into pcDNA3 vector by restriction enzyme digestion to generate a control plasmid. The control plasmids were linearized by restriction enzyme digestion, and inserts were cloned by seamless assembly using the In-Fusion Cloning kit (Takara). For dual-fluorescence reporter assays, constructs encoding 3× FLAG-miRFP and EGFP-3× HA separated by the P2A sequence were generated in the TOPGene backbone. For amino acid repeat reporters, repeat inserts were cloned upstream of a HiBiT spacer and the P2A sequence. For experiments using different linkers, one or three copies of a flexible GGGGS linker or a rigid EAAAK linker were inserted immediately upstream of the P2A sequence in constructs containing leucine repeats. All constructs were verified by Sanger sequencing (Cornell Biotechnology Resource Center) and whole-plasmid nanopore sequencing (Eurofins).

### Immunoblotting

Cells were rinsed with ice-cold DPBS and lysed in sodium dodecyl sulfate–polyacrylamide gel electrophoresis (SDS–PAGE) sample buffer (50 mM Tris–HCl, pH 6.8; 100 mM DTT; 2% SDS; 0.1% bromophenol blue; 10% glycerol) on ice, followed by heating at 95°C for 5 min. Proteins were separated by SDS–PAGE and transferred to PVDF membranes (Thermo Fisher). Membranes were blocked in TBST containing 5% nonfat milk for 1 h and incubated overnight at 4°C with primary antibodies in TBST with 5% milk. Primary antibodies used: anti-FLAG M2 (Sigma, F1804), anti-HA (Cell Signaling, 2367S), anti-eIF5A (Abcam, ab32407), anti-eRF1 (Santa cruz, sc-365686), and anti-β-Actin (Sigma, A5441). Following three washes in TBST (10 min each), membranes were incubated with HRP-conjugated secondary antibodies for 1 h at room temperature, washed, and visualized using enhanced chemiluminescence. For eRF1 degradation, cells were treated with SRI-41315 (5 µM, 24 h). The skipping score was calculated as the signal of Flag or HA, respectively, divided by the sum of the Flag or HA signal and the Flag–HA fusion product signal [13].

### Ribosome profiling

For plasmid reporters, 1 μg plasmid was transfected into 35-mm dishes for 24 h using Lipofectamine 3000 at a 1:1 ratio. Cells were washed with cold DPBS and lysed in polysome lysis buffer (10 mM HEPES, pH 7.4; 100 mM KCl; 5 mM MgCl₂; 100 μg/ml cycloheximide; 1% Triton X-100). Lysates were cleared by centrifugation at 20 000 × *g* for 10 min at 4°C and digested with RNase I (Ambion, 750 U per 100 *A*₂₆₀ units) at 4°C for 1 h. Total RNA was extracted using TRIzol LS (Invitrogen) for complementary DNA (cDNA) library construction.

### Ezra-seq and deep sequencing

Ribosome-protected fragments (25–35 nt) were resolved on a 15% polyacrylamide TBE–urea gel (Invitrogen) and visualized with SYBR Gold. Excised RNA fragments were eluted overnight in RNA elution buffer (300 mM sodium acetate, pH 5.2; 1 mM ethylenediaminetetraacetic acid; 0.1 U/μl SUPERase·In), cleared through Spin-X columns, and ethanol-precipitated. cDNA libraries were constructed using T4 PNK, Poly(A) polymerase, and custom Ezra enzyme, followed by ligation of 5′ adapters, streptavidin bead-based purification, and reverse transcription as described in the prior study [22]. Final cDNA was amplified with barcoded primers, size-selected (180 bp), quantified, pooled, and sequenced on an Illumina MiniSeq.

### Ribo-seq analysis

The reads were primarily mapped to a reporter sequence to obtain reporter-restricted ribosome density profiles using a custom code. The reporter sequences are available at https://github.com/usa0ri/P2A_2026/tree/main/reporter_sequences. Mapping to ribosomal A sites was performed by shifting the Ribo-seq read position 15 nt downstream from the 5′ end. The offset was determined from metagene profiles around start and stop codons for each read length. These analyses showed a 12-nt offset from the 5′ end to the start-codon-associated P site and a 15-nt offset from the 5′ end to the stop-codon-associated A site, consistently across the analyzed read lengths. For individual ribosome density plots, the raw read counts on P2A region were normalized to the counts on the first Pro codon of PGP motif. The P-score was defined as the ratio between the read counts on the GP codons over those on the PGP motif.

## Results

### Ribosome dynamics at the PGP motif during peptide-bond skipping

**Figure 1.**
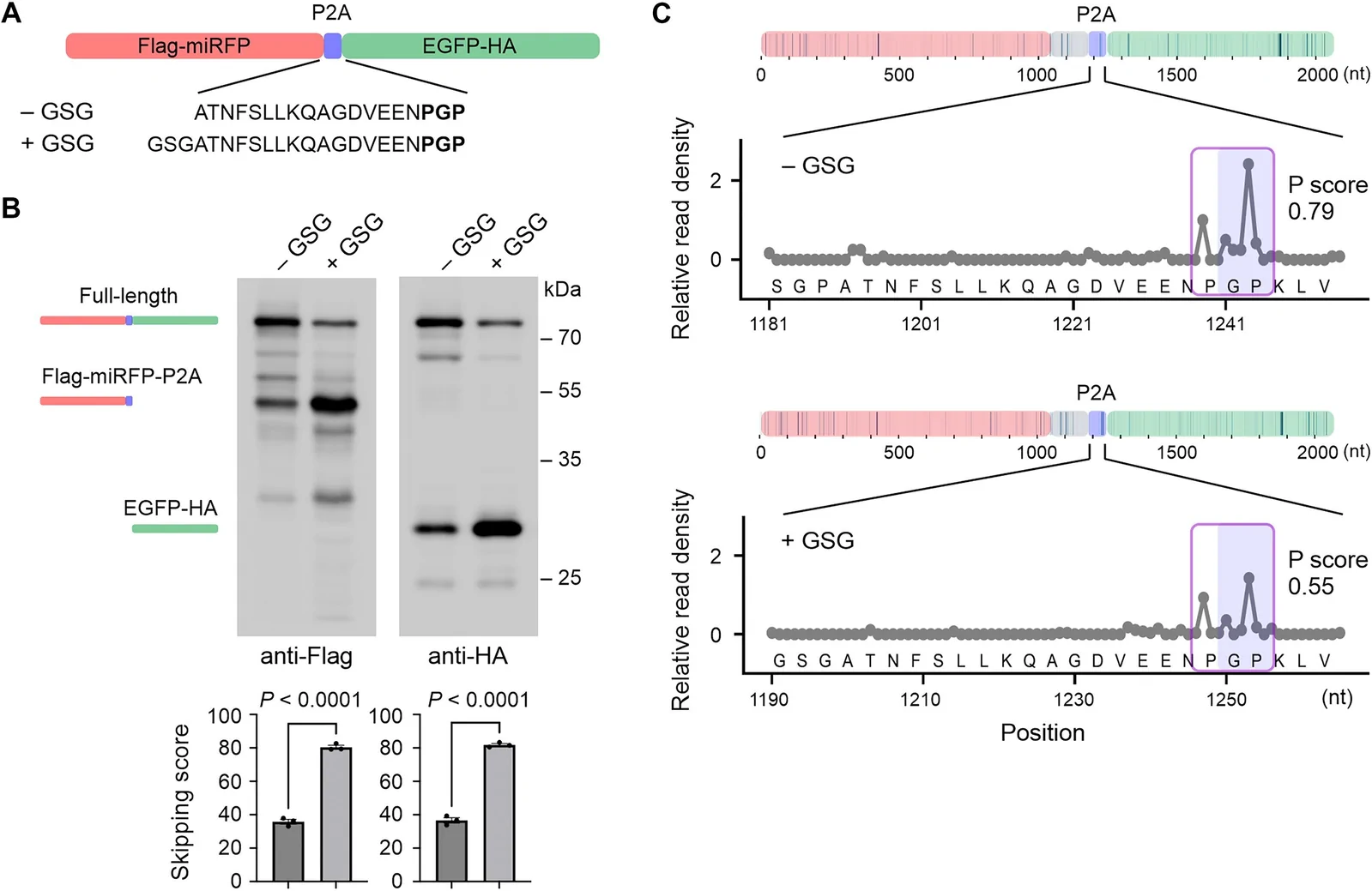
An upstream flexible linker modulates ribosome occupancy at the P2A junction. (**A**) Schematic of dual-reporter constructs containing FLAG-miRFP (red) and EGFP-HA (green) separated by P2A (blue), with or without an upstream GSG linker. (**B**) Immunoblotting of reporter products in HEK293 cells transfected with the indicated constructs. Skipping scores are shown as bar graphs. *n* = 3 biological replicates, error bar, ± SEM. *P*-values are from two-tailed unpaired Student’s *t*-test. (**C**) Distribution of normalized ribosome footprints across the reporter with or without upstream GSG linker. The read density of the P2A region is highlighted as a line plot. *P*-scores are calculated by the relative footprints at the terminal GP codons (blue shade) over the PGP motif (purple box).

To examine the relationship between ribosome dynamics at the PGP motif and peptide-bond-skipping efficiency, we engineered a dual-fluorescence reporter system for quantitative measurement of P2A-mediated recoding (Fig. 1A). The reporter comprises a 3× FLAG-tagged miRFP followed by a 3× HA tagged EGFP, separated by the P2A sequence. Skipping efficiency was quantified as the ratio of separate products to full-length protein using independent immunoblots with anti-Flag and anti-HA antibodies (Fig. 1B). Ribo-seq of transfected cells yielded sufficient coverage of the reporter (Fig. 1C), enabling codon resolution analysis of ribosome occupancy across the P2A region.

Insertion of the 19-aa P2A motif resulted in partial peptide-bond skipping (<40%), as reflected by the ratio of separated products to full-length proteins (Fig. 1B). Alignment of ribosome footprints to the P2A coding sequence revealed pronounced peaks at the PGP motif, with the strongest enrichment at the terminal Pro codon (Fig. 1C). Because A-site assignment was used for footprint mapping, this pattern indicates ribosome pausing at the junction between the P-site glycine and the A-site proline. Similar pausing has been documented for T2A sequences, albeit at lower resolution [23]. However, whether this pausing is causally linked to peptide-bond skipping remains unclear.

Previous studies showed that insertion of a flexible linker (e.g. GSG) immediately upstream of the 2A sequence enhances “stop-carry-on” activity [18]. Consistent with this, addition of a GSG linker increased skipping efficiency to >80% (Fig. 1B). Notably, ribosome footprint distributions across the reporter were highly similar between constructs. To control differences in sequencing depth, ribosome density was normalized to the first Pro codon of PGP. Using a *P*-score metric to estimate ribosome dwell time at the PGP junction, we found that the GSG linker reduced the *P*-score from 0.79 to 0.55 (Fig. 1C). Biological replicates of Ribo-seq obtained similar *P*-scores (Supplementary Fig. S1), excluding the variations associated with deep sequencing. These results indicate an inverse relationship between ribosome dwell time at the PGP motif and peptide-bond-skipping efficiency, suggesting that increased nascent chain flexibility promotes skipping by limiting ribosome pausing at the critical junction.

### eIF5A and eRF1 play limited roles in P2A-mediated peptide-bond skipping

**Figure 2.**
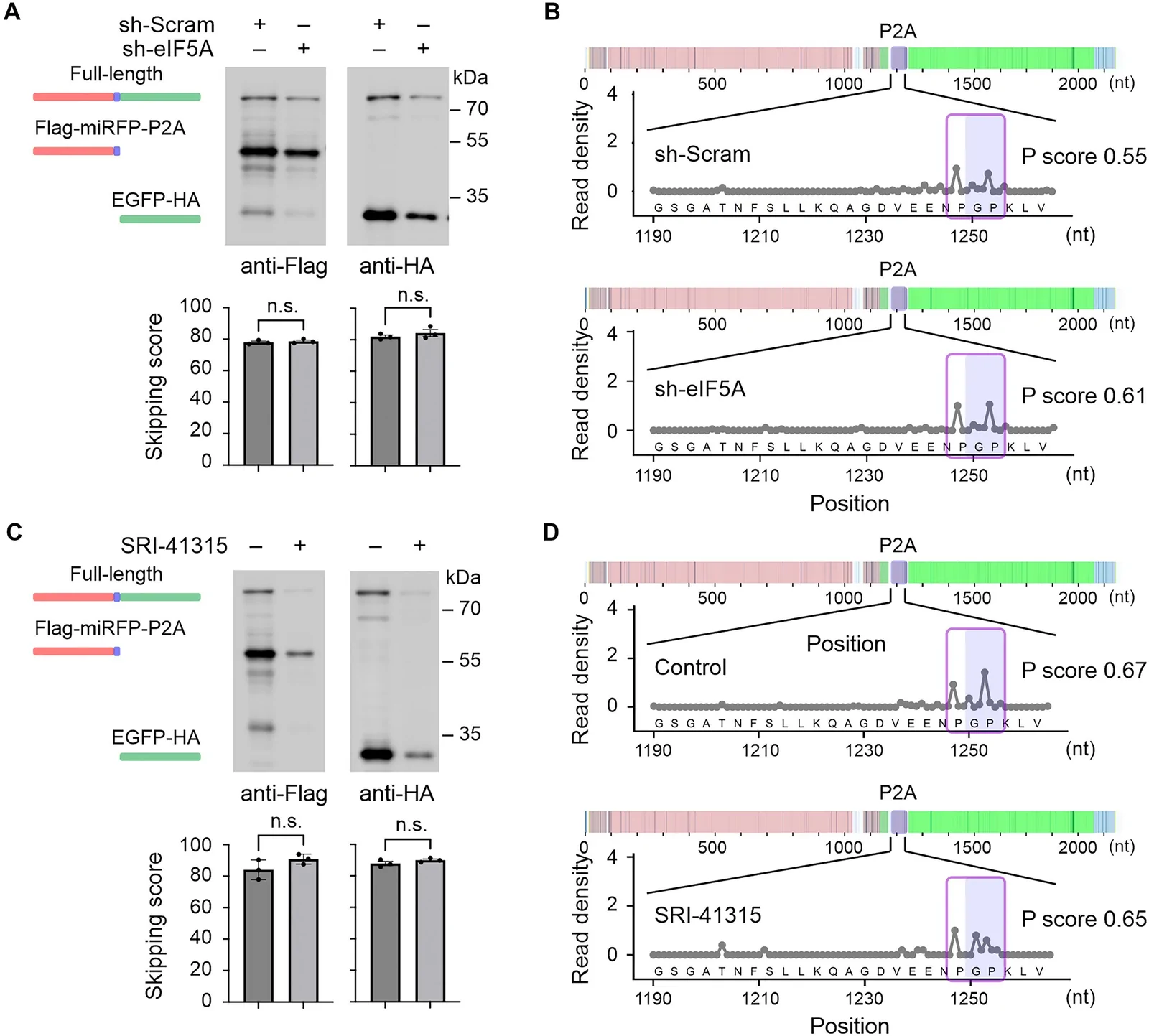
Negligible effects of eIF5A depletion and eRF1 degradation on P2A activity. (**A**) Immunoblotting of reporter products in control and eIF5A knockdown cells. The reporter construct contains GSG sequence before P2A. Skipping scores are shown as bar graphs. *n* = 3 biological replicates, error bar, ± SEM. (**B**) Distribution of normalized ribosome footprints across the reporter in transfected cells with or without eIF5A knockdown. The read density of the P2A region is highlighted as a line plot. *P*-scores are calculated by the relative footprints at the terminal GP codons (blue shade) over the PGP motif (purple box). (**C**) Immunoblotting of reporter products in control or cells treated with SRI-41315 (5 µM, 24 h). The reporter construct contains GSG sequence before P2A. Skipping scores are shown as bar graphs. *n* = 3 biological replicates, error bar, ± SEM. (**D**) Distribution of normalized ribosome footprints across the reporter in transfected cells with or without SRI-41315 treatment. The read density of the P2A region is highlighted as a line plot. *P*-scores are calculated by the relative footprints at the terminal GP codons (blue shade) over the PGP motif (purple box).

Peptide-bond formation involving proline is intrinsically slow because tRNA^Pro^ is both a poor peptidyl acceptor in the A-site and a suboptimal donor substrate in the P-site [15, 16]. However, slow decoding alone does not lead to peptide-bond skipping, as such events are rare during translation of poly(Pro) sequences. Poly(Pro) translation is facilitated by eIF5A, an elongation factor that binds to the ribosome E-site and modulates the PTC [16, 17]. To test whether eIF5A contributes to P2A-mediated peptide-bond skipping, we depleted eIF5A in HEK293 cells using shRNA (Supplementary Fig. S2A). As expected, eIF5A knockdown reduced global protein synthesis [16]. However, quantification of the P2A activity—measured as the ratio of separated products to the sum of full-length and separated products—revealed no significant change upon eIF5A knockdown (Fig. 2A). Consistently, Ribo-seq showed minimal effects on ribosome dwell time at the PGP motif, as indicated by similar *P*-scores (Fig. 2B). The same feature holds true when a reporter with extra upstream sequences was used (Supplementary Fig. S2B). These results suggest that, despite its critical role in poly(Pro) elongation, eIF5A does not contribute to P2A-mediated peptide-bond skipping.

2A-mediated peptide-bond skipping requires hydrolysis of peptidyl-tRNA in the P-site and subsequent release of the nascent chain. Early studies implicated translation termination factors in this process, based on increased readthrough upon partial depletion of eRF1 [10]. However, subsequent work suggested that 2A-mediated peptide separation occurs independently of canonical termination factors [13, 21, 24]. To directly assess the role of eRF1, we depleted eRF1 in HEK293 cells using the small-molecule degrader SRI-41315 [25, 26] (Supplementary Fig. S2C). Near-complete loss of eRF1 led to global inhibition of translation, as evidenced by reduced levels of full-length protein (Fig. 2C). Although a modest increase in the ratio of separated products to full-length protein was observed, this likely reflects altered protein abundance under translational repression rather than a change in intrinsic skipping efficiency. Ribo-seq confirmed global reduction in footprint density following SRI-41315 treatment. Importantly, ribosome occupancy at the terminal Pro codon of the PGP motif was not increased; instead, the *P*-score was slightly reduced (Fig. 2D). Together, these findings argue against a direct role of eRF1 in regulating P2A-mediated peptide-bond skipping.

### Differential effects of upstream amino acid repeats on P2A skipping efficiency

During 2A-mediated peptide-bond skipping, failed peptide-bond formation between the P-site glycine and A-site proline is coupled to nascent chain release [2]. Without hydrolysis of the peptidyl-tRNA at the P-site, the A-site tRNA^Pro^ cannot proceed with elongation (“carry-on”). The strong enhancement of 2A activity by upstream flexible linkers (e.g. GSG) suggests that efficient and timely release of the nascent chain from the ribosome exit tunnel is a key determinant of skipping. Beyond direct interactions with the exit tunnel, nascent chains engage in diverse co-translational processes upon emergence from the ribosome. Notably, extended regions upstream of 2A have been shown to inhibit 2A activity, yielding fusion proteins bearing N-terminal signal peptides that are translocated into the endoplasmic reticulum [27]. These observations raise the possibility that distal sequence features influence 2A skipping efficiency through long-range, co-translational events.

**Figure 3.**
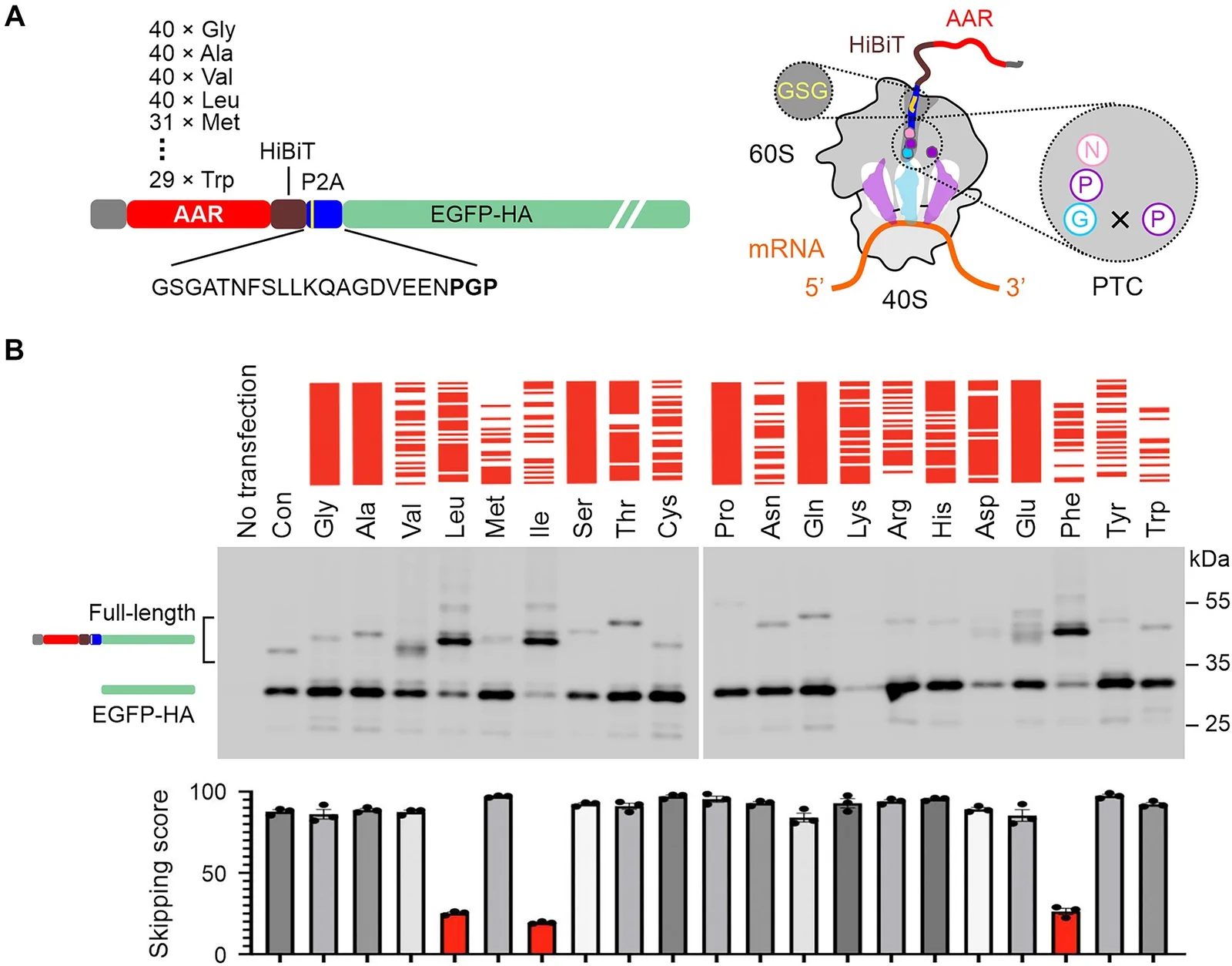
Differential effects of upstream amino acid repeats on P2A activity. (**A**) Schematic of reporter constructs containing amino acid repeat (AAR) inserts upstream of a HiBiT spacer and the P2A sequence. The right panel shows relative positions of nascent chain segments within the exit tunnel. (**B**) Immunoblot-based quantification of skipping efficiency for reporters containing the indicated amino acid repeat inserts. The top panel shows the occurrence of the amino acid within each AAR. Skipping scores are shown as bar graphs. *n* = 3 biological replicates, error bar, ± SEM.

To test this idea, we examined the impact of upstream amino acid repeats, which frequently serve as structural and functional elements in proteins. For example, leucine-rich repeats mediate protein–protein interactions [28], polyglutamine and polyalanine tracts contribute to transcriptional regulation and are associated with neurodegenerative and developmental disorders when expanded [29, 30], and low-complexity repeats can drive phase separation [31]. Endogenous repeat sequences were selected from the human genome (Supplementary Fig. S3) and positioned upstream of the P2A motif (Fig. 3A). To ensure that these repeats had emerged from the ribosome exit tunnel during decoding of the PGP motif, a spacer sequence encoding HiBiT (11 amino acids) was inserted between the repeats and the GSG linker. For each amino acid repeat, two top-ranked 20-codon repeats were concatenated to generate 40-codon inserts, except tryptophan-, phenylalanine-, and methionine-rich sequences due to limited tandem occurrence (Fig. 3A).

In HEK293 cells expressing these reporters, peptide-bond-skipping efficiency varied widely across 20 amino acid repeats, as quantified by the ratio of C-terminal EGFP to total protein (Fig. 3B). Small, flexible residues (e.g. glycine, alanine, and serine) had negligible effects on P2A-mediated peptide-bond skipping. As expected, poly(Lys) reduced overall protein output, consistent with ribosome stalling [32, 33], while acidic repeats (aspartate and glutamate) exhibited multiple bands of N-terminal species, a sign of translational discontinuation as reported previously [34–36]. However, peptide-bond skipping at the downstream PGP junction remained efficient. Notably, poly(Pro) had little effect on skipping efficiency, indicating that decoding at the PGP motif is mechanistically distinct from poly(Pro) elongation. Strikingly, hydrophobic repeats enriched in leucine, isoleucine, and phenylalanine strongly suppressed P2A activity, leading to accumulation of full-length fusion proteins and reduced EGFP production. These hydrophobic sequences also exhibit characteristic ladder-like polyubiquitination patterns on SDS–PAGE gel, indicative of co-translational quality control [37]. Together, these findings extend the previous report [27] by suggesting that nascent chain properties outside of the ribosome—particularly hydrophobic and associated quality control response—can allosterically inhibit 2A-mediated peptide-bond skipping within the PTC.

### Upstream hydrophobic residues negatively regulate P2A skipping

**Figure 4.**
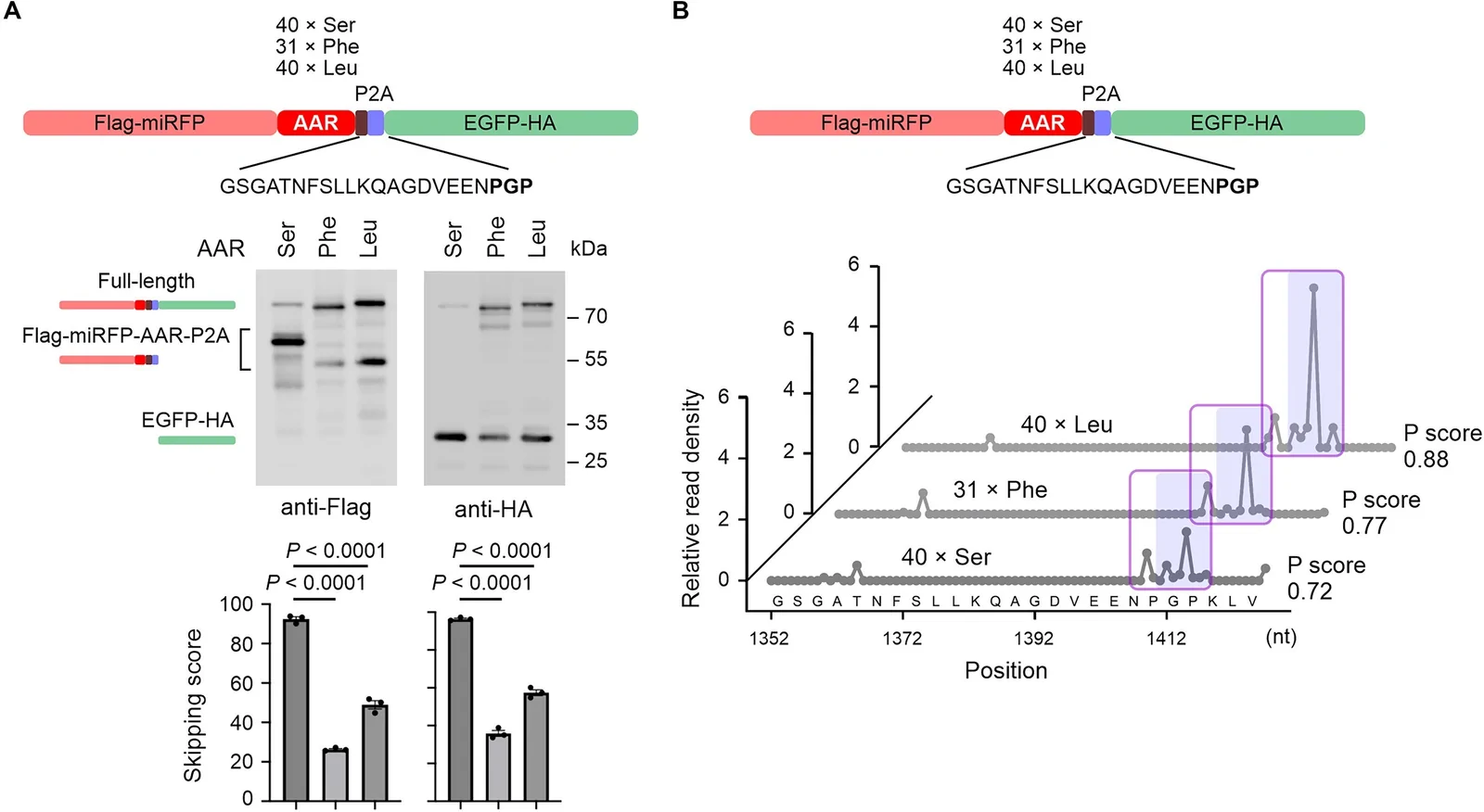
Upstream hydrophobic residues negatively regulate P2A skipping. (**A**) Schematic of reporter constructs containing serine-, phenylalanine-, or leucine-rich inserts upstream of P2A. The bottom panel shows immunoblotting of reporter products. Skipping scores are shown as bar graphs. *n* = 3 biological replicates, error bar, ± SEM. *P*-values are from two-tailed unpaired Student’s *t*-test. (**B**) Composite line plots show ribosome footprint distribution on the P2A region with phenylalanine-, or leucine-rich inserts. *P*-scores are calculated by the relative footprints at the terminal GP codons (blue shade) over the PGP motif (purple box).

To validate the inhibitory effect of upstream hydrophobic residues on 2A-mediated peptide-bond skipping, we constructed dual fluorescence reporters by including miRFP into the N-terminal region (Fig. 4A). This design leaves the inserted hydrophobic amino acid repeats in an internal region of the construct. Consistent with our initial observation, both phenylalanine and leucine repeats strongly suppressed P2A activity compared to the serine repeats. To directly assess ribosome dynamics, we conducted Ribo-seq in transfected cells. The presence of phenylalanine or leucine repeats markedly increased ribosome occupancy at the terminal proline codon of the PGP motif (Fig. 4B), indicating enhanced pausing at the peptide-bond-skipping junction. Notably, no additional pausing was detected across the HiBiT spacer and the 2A sequence, arguing against involvement of signal recognition particle-mediated targeting. We also examined whether the length of hydrophobic stretch matters in P2A activity (Supplementary Fig. S4). Intriguingly, even 10 consecutive leucine codons are sufficient to suppress downstream peptide-bond skipping. The leucine-rich sequences used here are largely derived from transmembrane domains, raising the possibility that co-translational interactions—such as chaperone engagement or membrane association—restrict nascent chain mobility [38–40]. Such constraints could impede nascent chain release from the P-site, prolong ribosome dwell time, and favor peptide formation over skipping.

**Figure 5.**
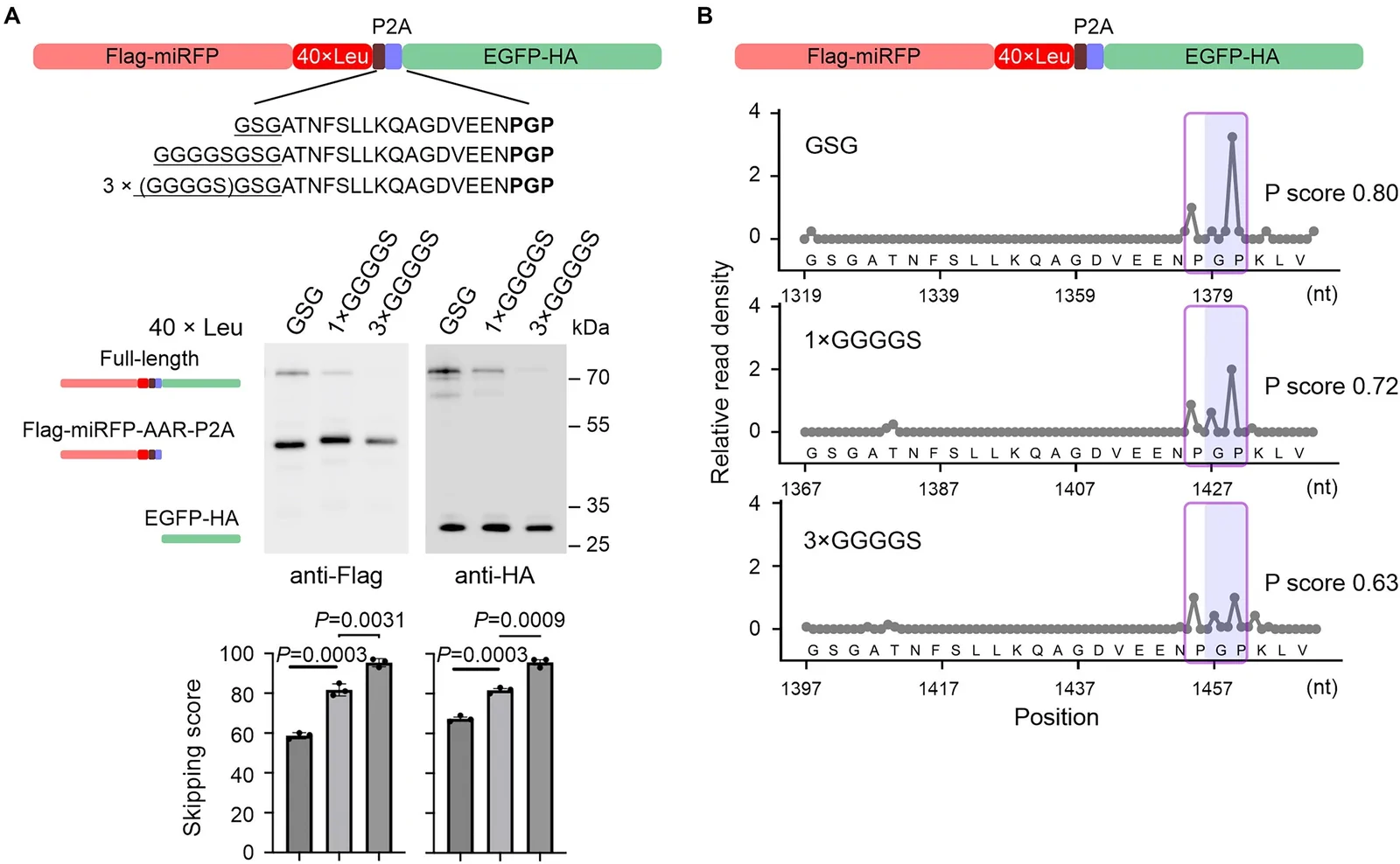
Opposing effects between hydrophobic residues and flexible linkers on P2A skipping. (**A**) Schematic of reporter constructs containing a leucine-rich insert together with increasing lengths of glycine-rich linkers upstream of P2A. The bottom panel shows immunoblotting of reporter products. Skipping scores are shown as bar graphs. *n* = 3 biological replicates, error bar, ± SEM. *P*-values are from two-tailed unpaired Student’s *t*-test. (**B**) Distribution of normalized ribosome footprints across the P2A region in reporters containing a leucine-rich insert together with increasing lengths of glycine-rich linkers upstream of P2A. *P*-scores are calculated by the relative footprints at the terminal GP codons (blue shade) over the PGP motif (purple box).

We next tested whether reduced nascent chain flexibility imposed by upstream hydrophobic segments could be counteracted by the flexible linker within the ribosome exit tunnel. To this end, we inserted one or three copies of a flexible GGGGS linker immediately upstream of the P2A sequence in constructs containing 40× leucine repeats (Fig. 5A). Introduction of these linkers restored the P2A-mediated peptide-bond skipping in a length-dependent manner, with the 3× GGGGS showing the strongest rescue. This suggests that increased spacing of nascent chains within the exit tunnel alleviates constraints imposed by distal hydrophobic sequences. Consistently, Ribo-seq revealed that the enhanced skipping efficiency correlated with reduced ribosome dwell time at the PGP motif, as evidenced by decreased *P*-scores (Fig. 5B). To distinguish whether the rescuing effect stems from prolonged distance or increased flexibility, we introduced more rigid EAAAK linker (Supplementary Fig. S5). To our surprise, those linkers also restored P2A skipping as effectively as—and in some configurations more effectively than—the flexible GGGGS linker. Given the stronger effect of 3× EAAAK, these results support a model in which co-translational properties of nascent chains exert long-range control over the PTC activity, establishing a feedback mechanism between co-translational events outside of the ribosome and ribosome recoding within the catalytic center.

## Discussion

2A-mediated peptide-bond skipping enables co-translational separation of sequentially encoded proteins without canonical termination [2, 10]. Previous studies using primer extension assays and ribosome profiling documented ribosome pausing at the conserved PGP junction and proposed that such pausing facilitates the recoding event [21, 23, 41]. Consistent with this view, recent structural work proposed that the upstream residues slow elongation to provide a temporal window for conformational arrangements within the PTC [13]. We observed a clear inverse relationship between peptide-bond-skipping efficiency and ribosome dwell time at the A-site Pro codon, regardless of the reporters used (Supplementary Fig. S6). This supports a kinetic model in which prolonged residence at the PGP motif increases the probability of peptide-bond formation between the P-site glycine and the A-site proline, thereby reducing skipping efficiency. However, our findings do not contradict the previously proposed model in which pausing at the PGP motif might be a prerequisite for skipping. Once pausing exceeds a certain threshold—as occurs with inhibitory upstream sequences—the prolonged dwell time is associated with reduced skipping efficiency, consistent with a shift in the kinetic competition back toward peptide-bond formation.

A central finding of this study is that the ribosome dynamics at the PGP motif are highly sensitive to upstream sequence context. The enhancement of skipping by flexible linkers (e.g. GSG) has been attributed to altered interactions between the nascent chain and the exit tunnel, potentially modulating PTC conformation. However, such a model would predict increased ribosome pausing, which is inconsistent with our observations. Instead, our results favor a model in which nascent chain flexibility promotes efficient hydrolysis of the peptidyl-tRNA at the P-site, thereby facilitating “stop-carry-on.” How peptide release occurs at a sense codon remains unresolved. Although spontaneous hydrolysis of peptidyl-tRNA has been proposed, its low intrinsic rate suggests that additional ribosome-intrinsic or nascent-chain-dependent mechanisms may enhance this reaction [14, 42].

Unexpectedly, the impact of upstream sequences extends beyond the ribosome exit tunnel. Hydrophobic segments, particularly leucine-rich sequences positioned distally upstream, strongly inhibit 2A-mediated peptide-bond skipping. Such sequences are known to engage co-translational pathways, including membrane targeting and chaperone binding [39, 43]. While we do not detect discrete pausing downstream of these regions, their emergence from the ribosome may impose physical constraints on the nascent chain, limiting the mobility and thereby hindering release from the PTC [44–46]. Consistent with this framework, hydrophobic sequences increase ribosome dwell time at the terminal Pro codon and reduce skipping efficiency, whereas insertion of flexible glycine-rich linkers alleviates these effects. These findings point to a long-range, mechanistic coupling between nascent chain properties outside the ribosome and catalytic events within the PTC. Direct structural information capturing the ribosome at the glycine–proline junction will be desirable to determine how upstream nascent-chain features influence PTC conformation, as current structural data do not fully resolve this transition state [13].

Although our analysis focuses on the P2A sequence, similar principles are likely applicable to other 2A elements, including recently identified class B 2A sequences [47]. Despite sequence divergence, these peptides share a conserved functional outcome, suggesting that modulation of ribosome dwell may represent a general mechanism underlying 2A-mediated translational recoding. Together, our results establish a kinetic and mechanistic framework linking nascent chain properties, ribosome dynamics, and translation recoding, with direct implications for the rational design of 2A-based expression systems.

## Supplementary Material

gkag875_Supplemental_File

## Data Availability

The sequencing data reported in this manuscript have been deposited in NCBI’s Gene Expression Omnibus under accession number GSE328587. Custom Python scripts used to analyze the sequencing data are available at GitHub and Zenodo under https://github.com/usa0ri/P2A_2026/tree/main and https://doi.org/10.5281/zenodo.19670703.
