# Supplementary material for: Ribosome dynamics at the conserved PGP motif governs 2A peptide-bond-skipping efficiency
Source: Nucleic Acids Res. 2026 Sep 8;54(17):gkag875. doi: 10.1093/nar/gkag875 (PMC13551032; doi:10.1093/nar/gkag875)
Supplement: Supplementary file 1 [file gkag875_supplemental_file.docx]

**Supplementary Figure 1. Biological replicates showing the effect of an upstream flexible linker on ribosome occupancy at the P2A junction.**

**(A)** Schematic of dual-reporter constructs containing FLAG-miRFP (red) and EGFP-HA (green) separated by P2A (blue), with or without an upstream GSG linker.
**(B)** Immunoblotting of reporter products in HEK293 cells transfected with the indicated constructs. Skipping scores are shown as bar graphs. *n* = 3 biological replicates, error bar, ± SEM. *P* values are from two-tailed unpaired Student’s *t*-test.

**(C)** Distribution of normalized ribosome footprints across the reporter with or without upstream GSG linker. The read density of the P2A region is highlighted as a line plot. P-scores are calculated by the relative footprints at the terminal GP codons (blue shade) over the PGP motif (purple box).

**Supplementary Figure 2. Effects of eIF5A depletion and eRF1 degradation on P2A activity.**

**(A)** Immunoblotting of HEK293 cells with or without eIF5A knockdown.

**(B)** Distribution of normalized ribosome footprints across the reporter in transfected cells with or without eIF5A knockdown. The read density of the P2A region is highlighted as a line plot. P-scores are calculated by the relative footprints at the terminal GP codons (blue shade) over the PGP motif (purple box).

**(C)** Immunoblotting of HEK293 cells treated with or without SRI-41315 (5 µM, 24 h).

**Supplementary Figure 3. List of nucleotide sequences selected for reporters harboring amino acid repeats**

**Supplementary Figure 4. Length effect of upstream hydrophobic residues on P2A skipping.**

Schematic of reporter constructs containing 10, 20, and 40 leucine-rich residues upstream of P2A. The bottom panel shows immunoblotting of reporter products. Skipping scores are shown as bar graphs. *n* = 3 biological replicates, error bar, ± SEM. *P* values are from two-tailed unpaired Student’s *t*-test.

**Supplementary Figure 5. Effects of different linkers on P2A skipping.**

Schematic of reporter constructs containing a leucine-rich insert together with increasing lengths of glycine-rich linkers or rigid EAAAK linkers upstream of P2A. The bottom panel shows immunoblotting of reporter products.

**Supplementary Figure 6.** A scatter plot shows the negative correlation between skipping scores and P scores, using data obtained from all P2A reporters.
